# T tube sinus tract duodenal fistula: a rare complication of postoperative choledochoscopy for treating retained intrahepatic stones

**DOI:** 10.1007/s00464-020-08057-7

**Published:** 2020-10-08

**Authors:** Jianying Lou, Hua Zhao, Wei Chen, Ji Wang

**Affiliations:** Department of Hepato-Pancreato-Biliary Surgery, The Second Affiliated Hospital, Zhejiang University School of Medicine, Hangzhou, Zhejiang China

**Keywords:** Intrahepatic stones, Choledochoscopy, T tube sinus tract, Duodenum, Fistula

## Abstract

**Backgrounds:**

Postoperative percutaneous choledochoscopy via T tube sinus tract is a common modality for treating retained intrahepatic stones in China. We report a rare complication of postoperative choledochoscopy for treating retained hepatolithiasis: T tube sinus tract duodenal fistula.

**Methods:**

From January 2003 to December 2018, intrahepatic duct stones with or without common bile duct stones were detected in 1031 patients. Fifteen of the 1031 patients with intrahepatic stones developed a T tube sinus tract duodenal fistula that was diagnosed by cholangiography and choledochoscopy.

**Results:**

The incidence of T tube sinus tract duodenal fistula in patients with retained intrahepatic stones being treated by postoperative choledochoscopy is 1.45% (15/1031) in this series. The chi-squared test showed that hypoalbuminemia (*P* = 0.003), long duration of T tube (*P* = 0.002), and high frequency of procedure (*P* = 0.008) might be associated with the occurrence of T tube sinus tract duodenal fistula. The logistic regression analysis demonstrated that hypoalbuminemia might be the independent risk factor for this special fistula (*P* = 0.037).

**Conclusions:**

Hypoalbuminemia, long time placement of T tube in situ and high frequency of procedure are probably the main causes of the T tube sinus tract duodenal fistula. Placement of T tube in correct way and improving nutritional status may be the key points to prevent the formation of T tube sinus tract duodenal fistula.

Hepatolithiasis or intrahepatic duct stones is prevalent in East Asia [1, 2]. In addition to choledochoscopy, various lithotripsy techniques and liver resection were widely used for treatment of intrahepatic duct stones, the clearance rate of intrahepatic stones has increased in recent years [3, 4]. Postoperative percutaneous choledochoscopy via the T tube sinus tract is a safe and effective approach for treating retained or residual intrahepatic stones [5]. The complications of percutaneous choledochoscopy such as fever (cholangitis), biliary duct bleeding, and T tube inadvertently dislodgement are usually mild and not life threatening. However, some rare complications related to percutaneous choledochoscopy are serious and may result in failure of retained stones extraction [6]. T tube sinus tract duodenal fistula due to long-term placement of T tube is a rare complication of postoperative percutaneous choledochoscopy that can lead to occlusion of distal channel of primary T tube sinus tract and failure to reach biliary duct for stones extraction. Here, we reported 15 cases of T tube sinus tract duodenal fistula that had not been described systematically by other authors.

## Methods

### Patients

From January 2003 to December 2018, a total of 1031 patients with retained intrahepatic duct stones after surgery underwent percutaneous choledochoscopy sessions by two surgeons at the 2nd Affiliated Hospital of Zhejiang University School of Medicine in Hangzhou, China. Patients with hepatolithiasis included in this series had the history of common bile duct (CBD) explorations or choledochotomies and intraoperative stones extractions (*n* = 725), liver resections (*n* = 263), choledocho- or hepaticojejunostomies (*n* = 31), and choledocho-cutaneous-jejunostomies (*n* = 12). All patients underwent cholecystectomies.

### Procedures

A firm fibrous T tube sinus tract will be established after the T tube was placed in situ for 6 ~ 8 weeks postoperatively. So the initial postoperative choledochoscopy was performed at least 6 weeks after the operation in all 1031 patients. A flexible fiber or electronic choledochoscope was inserted through the T tube sinus tract after removal of the T tube for stones extraction choledochoscopically. The T tube will be reinserted into CBD after the stone extraction procedure for next session when the retained stones were not completely removed. Electrohydraulic lithotripsy (EHL), ballistic or laser lithotripsy was used for fragmentation of the complicated stones such as the large, impacted stones that cannot be retrieved by a basket or the calculi that was beyond the biliary stricture. When clearance of the retained intrahepatic stones was achieved choledochoscopically, the T tube was removed after further examination by cholangiography and ultrasound. Majority of the postoperative percutaneous choledochoscopies are performed in daycare unit in our hospital without any anesthesia because of low complication occurrence rate and minimal invasion.

### Statistical analysis

SPSS 19.0 software (SPSS, Inc., Chicago, IL, USA) was used to perform statistical analysis. The chi-squared test was used to examine the association between fistula and various patients’ clinical parameters. Logistic regression analysis was carried out to identify the potential risk factors of fistula. *P* value < 0.05 was considered to be statistical significance.

## Results

Intrahepatic duct stones with or without CBD stones were detected in 1031 patients (469 men, 562 women) ranging in age from 18 to 93 years (mean 56 years) including 578 cases with left intrahepatic duct stones, 225 cases with right intrahepatic duct stones, and 228 cases with stones in both sides.

**Fig. 1:**
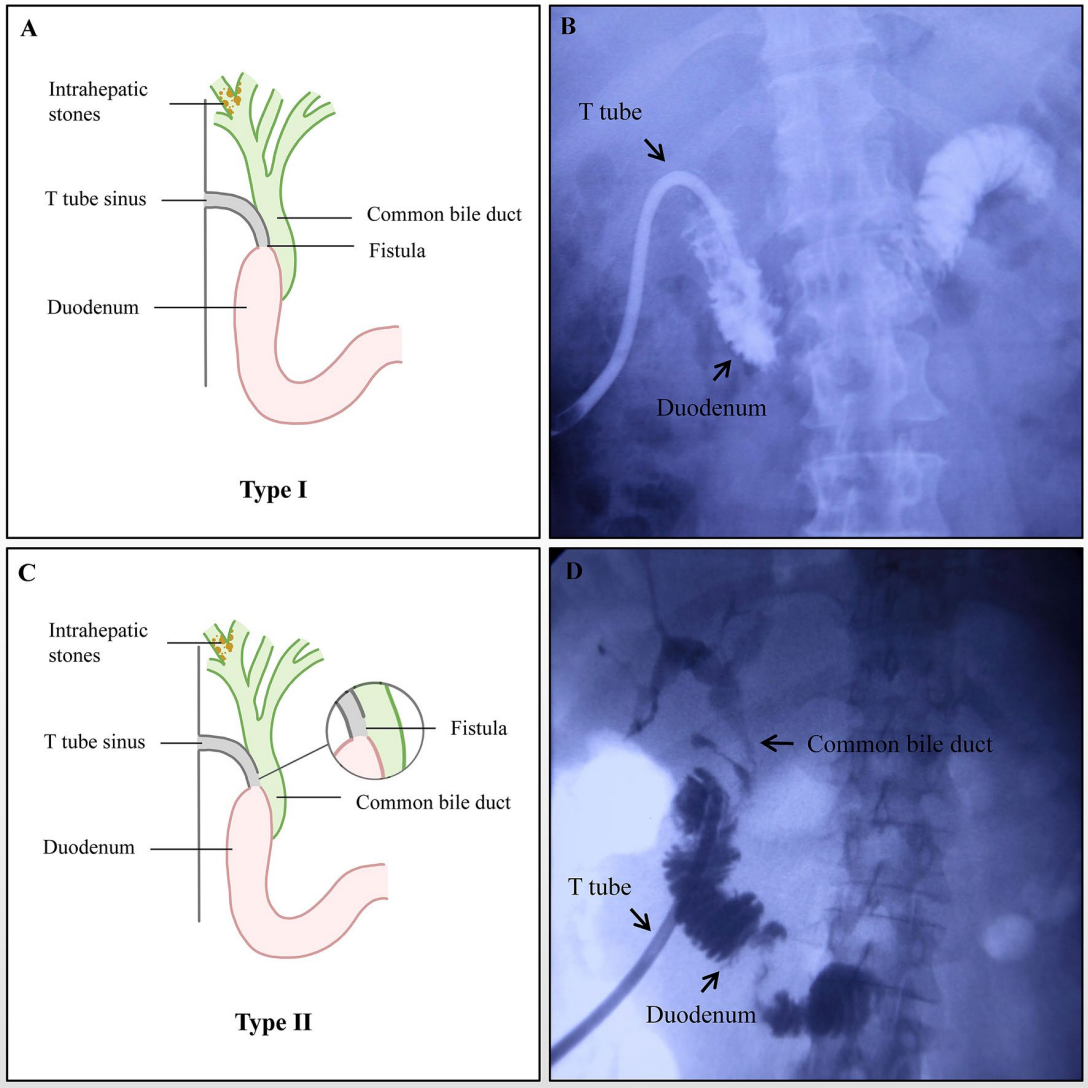
Type I fistula **A, B** The direct passage of contrast material into duodenum but no biliary tree was showed by T tube cholangiography. Type II fistula **C, D** Instead of biliary tree, the duodenal loop was first showed in fluoroscopy when the contrast material was infused through T tube and then the common bile duct (CBD) and intrahepatic duct was developed

**Fig. 2:**
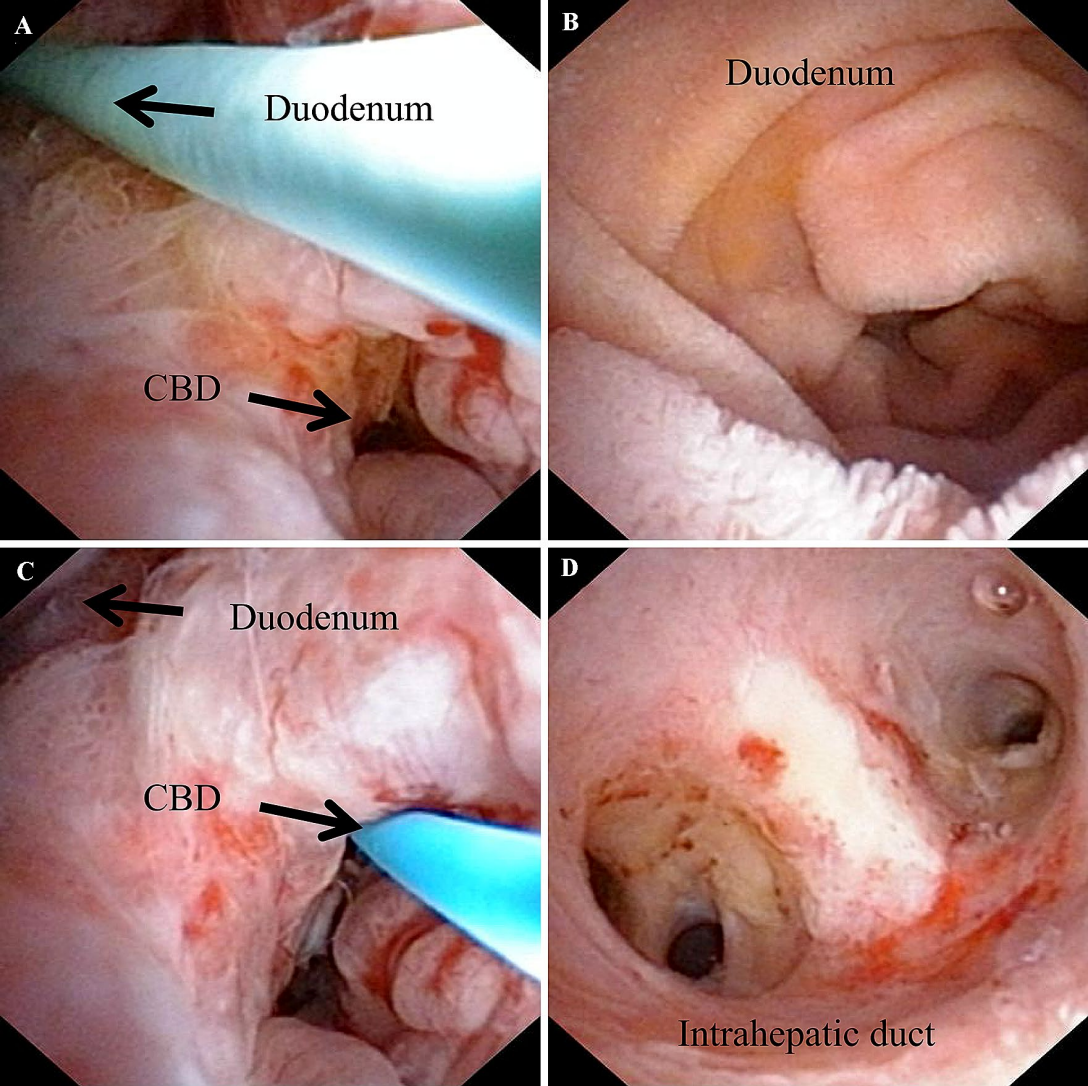
The choledochoscope can get into both sides of duodenum (**A, B**) and common bile duct (**C, D**) in type II fistula

Fifteen of the 1031 (1.45%) patients with intrahepatic stones included in this series developed a T tube sinus tract duodenal fistula that was diagnosed by cholangiography and choledochoscopy. The fistulas were not observed during the previous procedure and the T tubes were unintentionally inserted into duodenum through the T tube tract duodenal fistula channel in 9 patients. After a mean duration of 2.3 weeks (with a range of 1 to 4 weeks), occlusions of the distal channel of T Tube were developed and resulted in failure to get access to CBD for further stones extraction choledochoscopically. The direct passage of contrast material into duodenum but no biliary tree was showed by T tube cholangiography in these 9 patients because of the blockage of the distal T tube sinus tract. We classified this kind of fistula as type I (Fig. 1A and B). The fistulas were identified by choledochoscope during the stones extraction procedure in other 6 patients who all underwent a cholangiography before the diagnosis was established. Instead of biliary tree, the duodenal loop was first showed in fluoroscopy when the radiographic contrast was infused through T tube in patients with T tube sinus tract duodenal fistula. We classified this kind of fistula as type II (Fig. 1C and D). The choledochoscope can get into both sides of duodenum and CBD (Fig. 2) and finished the stones extraction. A guide wire was introduced into CBD guided by choledochoscope and followed by insertion of the T tube along the guide wire. All retained stones were cleared by choledochoscopy in these 6 patients without other complications. No specific symptoms were presented in these 15 patients with T tube sinus tract duodenal fistula. The presence of food debris or chyme mixed with bile juice in T tube was a common manifestation in some patients due to dislocation of the T tube into the duodenum after stones extraction choledochoscopically.

All of these 15 patients (4 men, 11 women) ranging in age from 52 to 75 years had retained intrahepatic stones after open surgeries. T tubes had been placed for a range of 8 weeks to 11 months in these 15 patients. The frequency of postoperative percutaneous choledochoscopic procedure for stones extraction ranged from 2 to 15 times. The chi-squared test was used between patients with and without fistula and the results showed that hypoalbuminemia (*P* = 0.003), long duration of T tube (*P* = 0.002), and high frequency of procedure (*P* = 0.008) might be associated with the occurrence of T tube sinus tract duodenal fistula. The logistic regression analysis demonstrated that hypoalbuminemia might be the independent risk factor for this special fistula (*P* = 0.037) (Table 1).

**Table 1:** Univariate analysis and logistic regression analysis of 7 clinical parameters between fistula group and non-fistula group

	Fistula group	Non-fistula group	Univariate analysis		Logistic regression analysis	
	(*n* = 15)	(*n* = 1016)	*χ*^2^	*P* value	HR (95% CI)	*P* value
Gender			0.148	0.787		
Male	4	318				
Female	11	698				
Age (years)			0.199	0.796		
> 60	8	476				
< 60	7	540				
Albumin (g/L)			7.645	0.003	3.290 (1.075–10.069)	0.037
< 30	7	188				
> 30	8	828				
Bilirubin (mmol/L)			0.866	0.416		
< 34	12	699				
> 34	3	317				
Diabetes			0.125	1		
Yes	4	314				
No	11	702				
T tube duration			11.075	0.002	2.371 (0.477–11.790)	0.291
> 3 months	11	331				
< 3 months	4	685				
Frequency of procedure			8.009	0.008	2.415 (0.575–10.145)	0.229
> 5 times	9	274				
< 5 times	6	742				

*HR* hazard ratio, *CI* confidence interval

All cutaneous fistulas healed ranged from 3 to 7 days after removal of the T tubes. Nine patients with type I fistula had residual stones in this series. Four patients with residual intrahepatic stones underwent operations to reconstruct the sinus because of recurrent cholangitis. Other five patients without any symptoms chose to regular examinations.

## Discussion

Liver resection and intraoperative choledochoscopy are effective modalities for treating patients with hepatolithiasis that is prevalent in China [7, 8]. The presence of biliary duct strictures, liver fibrosis or atrophy, and multiple focal microabscesses are indications for liver resection that has been widely performed for treatment of hepatolithiasis in our department in these two decades. Although a combination of liver resection and intraoperative choledochoscopy was used, some patients with bilateral stones and multiple stones in one lobe still need postoperative choledochoscopy for clearance of retained stones [9]. According to our experience and other authors, postoperative choledochoscopy is still an effective approach to remove the residual intrahepatic stones and raise the clearance rate [10].

Cholangitis, biliary duct bleeding, accidental loss of sinus tract, and rupture of the tract are the most common complications of percutaneous choledochoscopy. T tube tract duodenal fistula was first described as fistula formation between sinus and duodenum in 1980 [11]. The occurrence rate of T tube sinus tract duodenal fistula in his series was 0.34% (1/292), but no detailed conditions were described in this paper. Other authors sporadically reported similar complications that were described as small bowel fistula [6, 12].

Postoperative percutaneous choledochoscopy is a simple and safe procedure, but time-consuming. T tube needs to be replaced at the end of each session of choledochoscopy. The procedure is usually repeated every 1 to 2 weeks until all intrahepatic duct stones have been extracted. In our series, we suggested that long-term placement of T tube in situ and high frequency of procedure might compress and erode the wall of the duodenum. Hypoalbuminemia was possibly risk factor of T tube sinus tract duodenal fistula formation. Therefore, if the T tube needs to be placed for more than 3 months, especially in patients with hypoalbuminemia, we should keep in mind that a T tube sinus tract duodenal fistula tends to be developed. When the T tube sinus tract duodenal fistula is not found during the procedure, the replaced T tube will be easily inserted into the duodenum, which lead to the occlusion of distal T tube tract and failure to get access to CBD in the next stones extraction session.

According to our experience on management of this rare and serious complication, some prophylactic measures can be taken for the prevention of a T tube tract duodenal fistula. Firstly, hepatectomy, which not only removes calculi and strictures but also eliminates the risk of intrahepatic cholangiocarcinoma and intraoperative choledochoscopy are first choice for management of complicated intrahepatic stones especially for patients with biliary duct strictures, liver fibrosis or atrophy, and impacted big stones [7, 13, 14]. Ultrasound-guided intraoperative choledochoscopy could be a good option to remove stones as many as possible during the operation and reduce the difficulty of postoperative procedures [8]. Secondly, at the time of surgery, T tube should be placed in a correct way and be taken out straightly and vertically through the abdominal wall to make sure the intraperitoneal section of the T tube does not compress the wall of the duodenum or other intestines. In some situations, the omentum can be used as a good cushion to prevent the duodenum from being eroded by the T tube.

Treatment of T tube sinus tract duodenal fistula is relatively simple. When the T tube was inserted into the duodenum through the fistula connecting the middle part of the T tube sinus tract with the duodenum and the distal T tube sinus tract was occluded, it is difficult to reconstruct the primary T tube sinus tract using percutaneous approach. So if the diagnoses of T tube sinus tract duodenal fistula and occlusion of the distal T tube sinus tract are established, there are no options except for removal of the T tube and occlusion of the fistula. Interestingly, the T tube sinus tract duodenal fistula, unlike the conventional duodenal or intestinal fistula, is easy to heal in short time without further treatment. The residual stones in biliary tree can be removed by further endoscopic sphincterotomy or open surgery depending on the location and the quantity of the calculi. When the T tube sinus tract duodenal fistula was identified at the time of postoperative choledochoscopic stones extraction, a guide wire should be inserted into the bile duct through the primary T tube sinus tract guided by choledochoscope at the end of the procedure and then the T tube replaced along the guide wire to ensure the continuity and intact of the primary T tube sinus tract.

In sum, we presented 15 patients with intrahepatic stones who developed a T tube sinus tract duodenal. Long duration of T tube and high frequency of procedure might be associated with its occurrence and hypoalbuminemia was the independent risk factor for this special fistula.
